# The Balance of MU-Opioid, Dopamine D2 and Adenosine A2A Heteroreceptor Complexes in the Ventral Striatal-Pallidal GABA Antireward Neurons May Have a Significant Role in Morphine and Cocaine Use Disorders

**DOI:** 10.3389/fphar.2021.627032

**Published:** 2021-03-15

**Authors:** Dasiel O. Borroto-Escuela, Karolina Wydra, Ramon Fores-Pons, Lakshmi Vasudevan, Wilber Romero-Fernandez, Małgorzata Frankowska, Luca Ferraro, Sarah Beggiato, Minerva Crespo-Ramirez, Alicia Rivera, Luisa L. Rocha, Miguel Perez de la Mora, Christophe Stove, Małgorzata Filip, Kjell Fuxe

**Affiliations:** ^1^ Department of Neuroscience, Karolinska Institutet, Biomedicum, Stockholm, Sweden; ^2^ Department of Drug Addiction Pharmacology, Maj Institute of Pharmacology, Polish Academy of Sciences, Kraków, Poland; ^3^ Laboratory of Toxicology, Department of Bioanalysis, Faculty of Pharmaceutical Sciences, Ghent University, Ghent, Belgium; ^4^ Department of Life Sciences and Biotechnology, University of Ferrara, Ferrara, Italy; ^5^ Department of Medical, Oral and Biotechnological Sciences, University of Chieti-Pescara, Chieti, Italy; ^6^ Instituto de Fisiología Celular, Universidad Nacional Autónoma de México, Mexico City, Mexico; ^7^ Department of Cell Biology, University of Malaga, Instituto de Investigación Biomédica (IBIMA), Malaga, Spain; ^8^ Pharmacobiology Department, Center for Research and Advanced Studies, Mexico City, Mexico

**Keywords:** G protein-coupled receptor, mu opioid receptor, dopamine D2 receptor, adenosine A2A receptor, morphine use disorder, cocaine use disorder, oligomerization, morphine

## Abstract

The widespread distribution of heteroreceptor complexes with allosteric receptor-receptor interactions in the CNS represents a novel integrative molecular mechanism in the plasma membrane of neurons and glial cells. It was proposed that they form the molecular basis for learning and short-and long-term memories. This is also true for drug memories formed during the development of substance use disorders like morphine and cocaine use disorders. In cocaine use disorder it was found that irreversible A2AR-D2R complexes with an allosteric brake on D2R recognition and signaling are formed in increased densities in the ventral enkephalin positive striatal-pallidal GABA antireward neurons. In this perspective article we discuss and propose how an increase in opioid heteroreceptor complexes, containing MOR-DOR, MOR-MOR and MOR-D2R, and their balance with each other and A2AR-D2R complexes in the striatal-pallidal enkephalin positive GABA antireward neurons, may represent markers for development of morphine use disorders. We suggest that increased formation of MOR-DOR complexes takes place in the striatal-pallidal enkephalin positive GABA antireward neurons after chronic morphine treatment in part through recruitment of MOR from the MOR-D2R complexes due to the possibility that MOR upon morphine treatment can develop a higher affinity for DOR. As a result, increased numbers of D2R monomers/homomers in these neurons become free to interact with the A2A receptors found in high densities within such neurons. Increased numbers of A2AR-D2R heteroreceptor complexes are formed and contribute to enhanced firing of these antireward neurons due to loss of inhibitory D2R protomer signaling which finally leads to the development of morphine use disorder. Development of cocaine use disorder may instead be reduced through enkephalin induced activation of the MOR-DOR complex inhibiting the activity of the enkephalin positive GABA antireward neurons. Altogether, we propose that these altered complexes could be pharmacological targets to modulate the reward and the development of substance use disorders.

## Introduction

The adenosine A2A receptor (A2AR)-dopamine D2 receptor (D2R) heteroreceptor complexes and their allosteric receptor-receptor interactions in the ventral striatal-pallidal GABA antireward neurons are of high relevance for understanding cocaine reward and cocaine use disorder (Trifilieff et al., 2011; Pintsuk et al., 2016; Borroto-Escuela et al., 2018a; Borroto-Escuela et al., 2018b; Wydra et al., 2020; Zhu et al., 2020). This pathway connects the ventral striatum, mainly nucleus accumbens, with the ventral pallidum and modulates the glutamate drive to the prefrontal cortex from the mediodorsal thalamic glutamate neurons (Groenewegen, 1988). It takes place via the ventral pallidal GABA pathway to the mediodorsal thalamic nucleus (Fuxe et al., 2008). The ventral striatal-pallidal GABA anti-reward pathway is linked to aversion and punishment as found in learning experiments using optogenetic techniques (Kravitz et al., 2013; Soares-Cunha et al., 2016). The concept of antireward neurons was introduced in the work of Koob and Le Moal (Everitt et al., 2008; Koob and Le Moal, 2008). It was found in rat that cocaine self-administration may produce pathological A2AR-D2R complexes having a strong and long-lasting brake on D2R recognition and signaling (Pintsuk et al., 2016; Borroto-Escuela et al., 2017b; Borroto-Escuela et al., 2018a; Borroto-Escuela et al., 2018b; Borroto-Escuela et al., 2020b). It appears to result in a maintained state of antireward and aversion since the D2R in these complexes cannot signal and no longer inhibit the activity of these antireward neurons leading to the development of cocaine use disorders. A new treatment of cocaine use disorder may therefore be represented by receptor interface-interfering peptides that impede the formation of these heteroreceptor complexes (Borroto-Escuela et al., 2018b).

**FIGURE 1:**
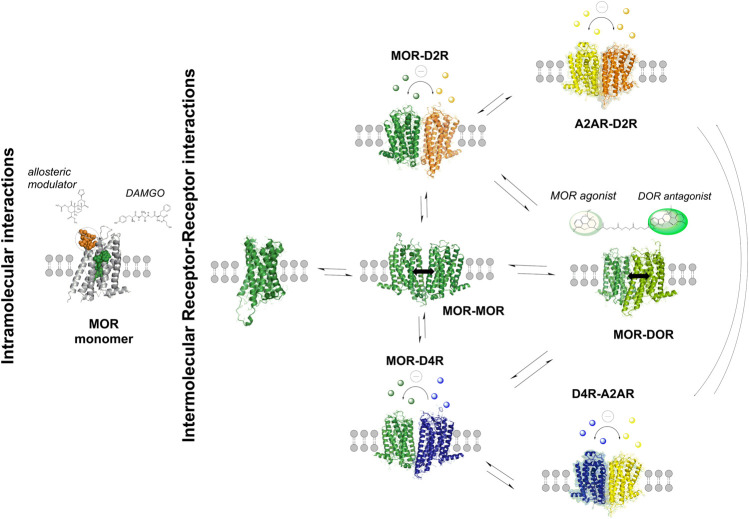
A propose model on the existence of MOR, D2R and A2AR homo-and heteroreceptor complexes in balance with each other in the ventral striatal-pallidal GABA antireward neurons, shown as dimers. The MOR monomer and its intramolecular interactions are shown in the left panel. The orthosteric MOR receptor binding site is indicated in yellow to which e.g., the MOR agonist DAMGO (in green) can bind. The allosteric MOR binding site is shown in yellow. The intermolecular receptor-receptor interactions in adenosine, dopamine and opioid homo-and heteroreceptor complexes, shown as dimers, are indicated in the right panel. The balance between the oligomers is indicated by two arrows perpendicular to each other. It should be noted that A2R-dopamine D4 receptor (D4R) heteroreceptor complexes also exist in the rat forebrain, as demonstrated by the proximity ligation assay (Borroto-Escuela and Fuxe, 2019; Borroto-Escuela et al., 2020a). They were found in substantial densities in the prefrontal cortex, especially in the somatic membrane of the internal pyramidal cell layer V, and in the dorsal hippocampus, especially in the pyramidal cell layer (Borroto-Escuela and Fuxe, 2019). They also exist in high densities in the nucleus accumbens and dorsal striatum including both striosome and matrix compartments. Moreover, the D4Rs in the cortical regions have a role in cognition (Furth et al., 2013) that may be modulated by A2AR through the demonstrated A2AR-D4R heteroreceptor complexes in the prefrontal cortex and the hippocampus. Whether these receptor complexes have a role in cocaine and morphine use disorders, requires further analyses.

The first indication for the existence of D2R-μ opioid receptor (MOR) complexes in the central nervous system (CNS) was obtained by Dai and colleagues in 2016 who demonstrated coimmunoprecipitation of MOR and D2R in the spinal cord of mice (Dai et al., 2016). Heterodimerization between MOR and D2R, ectopically expressed in HEK 293T and HeLa cells, was supported via several techniques such as BRET^1^, FRET and functional complementation (Vasudevan et al., 2019) (Figure 1). These findings are of interest since it shows that two major receptors linked to substance use disorders can physically interact. It should be noted that a D2R antagonist can diminish morphine tolerance in mice spinal cord (Dai et al., 2016).

Taken together, these results open the possibility that A2AR-D2R [observed in rat (Borroto-Escuela et al., 2013b; Borroto-Escuela et al., 2017b; Feltmann et al., 2018), mice (Trifilieff et al., 2011) and human (Zhu et al., 2020)] and D2R-MOR [observed in rat (Vasudevan et al., 2019)] heteroreceptor complexes can both exist in the ventral striatal-pallidal GABA antireward neurons in balance with their respective monomers/homomer complexes (Borroto-Escuela et al., 2016; Feltmann et al., 2018; Moller et al., 2020). These neurons are well-known to contain enkephalins that can be released both from their nerve terminals and from soma-dendritic regions (Mongi-Bragato et al., 2016). Thus, there should be a balance between these two types of D2R heteroreceptor complexes in the antireward neurons. In addition, they can also contain MOR-δ opioid receptor (DOR) heteroreceptor complexes which have been observed in rodents and are important targets for morphine [see e.g., (Gomes et al., 2000; Gupta et al., 2010; Borroto-Escuela et al., 2013c; Kabli et al., 2014; Erbs et al., 2016; Derouiche and Massotte, 2019)] (Figure 1). There should also exist a balance between these two types of MOR complexes present in the antireward neurons.

The ventral striatal-pallidal GABA antireward neurons, also known as D2R positive medium spiny neurons of the nucleus accumbens (Kupchik and Kalivas, 2017), express both D2R homo and heteroreceptor complexes (Feltmann et al., 2018). However, the possibility should be considered that D2R-MOR heteroreceptor complexes may only exist in a proportion of the ventral striatal-pallidal GABA antireward neurons. This may also hold true for the A2AR-D2R and MOR-DOR heteroreceptor complexes. In the case of MOR-DOR heteroreceptor complexes, however, we propose that MOR-DOR complexes can also be formed in the GABA antireward neurons possessing D2R-MOR heteroreceptor complexes. This aspect of integration of multiple heteroreceptor complexes remains to be covered in future work. In the current perspective article, we only discuss the integration of multiple heteroreceptor complexes on the basis that they can be formed in the same ventral striatal-pallidal GABA antireward neuron but in a dynamic balance that can be critically altered e.g., in morphine use disorder.

One question that comes out is whether A2AR-D2R complexes may also play a significant role in animal models of morphine use disorder e.g., related to changes in the balance with D2R-MOR complexes and their balance with MOR-DOR complexes. The formation of the heteroreceptor complexes is dependent on several factors like the density of the two receptor protomers and the affinity of the two receptor protomers for each other. The GPCR complexes can also contain ion channel receptors, receptor tyrosine kinases (RTKs), sets of G protein interacting proteins and/or transmitter transporters increasing their integrative capability (Liu et al., 2000; Lee and Liu, 2004; Flajolet et al., 2008; Borroto-Escuela et al., 2012; Borroto-Escuela et al., 2013a). The presence of adaptor proteins in the receptor complex, like sigma 1 receptor, RAMPs, can also be a relevant factor and receptor agonists also modulate the receptor complexes through conformational changes leading to antagonistic or enhancing receptor-receptor interactions (Fuxe et al., 2009). To/day, there is a lack of knowledge on the stoichiometry of the participating receptor protomers in MOR heteroeceptor complexes. However, super-resolution imaging methods (Owen et al., 2013) and spatial intensity distribution analysis (Ward et al., 2015) have been developed which can be used to determine the stoichiometry in cellular models. An important issue is to consider that different receptor complexes can also compete for the same receptor protomer since they exist in balance with each other (Fuxe et al., 2014a; Fuxe et al., 2014b; Borroto-Escuela et al., 2015; Borroto-Escuela et al., 2016; Borroto-Escuela et al., 2017a). This is the case of a heteromer and its corresponding homomers and different heteromers sharing one or two receptor protomers (Borroto-Escuela et al., 2016; Borroto-Escuela et al., 2018a) (Figure 1).

The measure of the balance between these complexes in natural system without genetic modification or overexpression remains a challenge. However, the use of *in situ* Proximity Ligation Assay (Trifilieff et al., 2011; Borroto-Escuela et al., 2013b; Borroto-Escuela et al., 2016; Fuxe and Borroto-Escuela, 2018; Zhu et al., 2020) or a combination of this method with others (e.g., microscale thermophoresis, biophysical TR-FRET between protomer ligands (Albizu et al., 2010; Ciruela et al., 2014), and chemical crosslinking co-immunoprecipitation followed by SDS-PAGE/MSMS[Borroto-Escuela et al., 2011; Odagaki and Borroto-Escuela, 2019)] provide unambiguous and accurate information.

In this perspective we review literature data on the effects of A2AR agonists and antagonists in morphine self-administration and morphine withdrawal. We will then evaluate to which extent such alterations can be explained by changes in the balance of the A2AR-D2R, D2R-MOR and MOR-DOR heteroreceptor complexes in the ventral striatal-pallidal GABA antireward neurons. As two major receptors involved in addiction, D2R and MOR can form a receptor heteromer (Vasudevan et al., 2019) which likely exists in cocaine and morphine use disorders. We propose that changes in D2R-MOR heteroreceptor complexes may be a marker for development of morphine use disorders. So far, there are indications that the interaction in this heteromer may be antagonistic (Vasudevan et al., 2019). Also, the dopamine D2R antagonist appeared to disrupt the receptor-receptor interaction, leading to a reduction of morphine tolerance (Dai et al., 2016).

## Modulation of Maintenance of Morphine Self-Administration by Receptor Protomer Agonists and Antagonists

**FIGURE 2:**
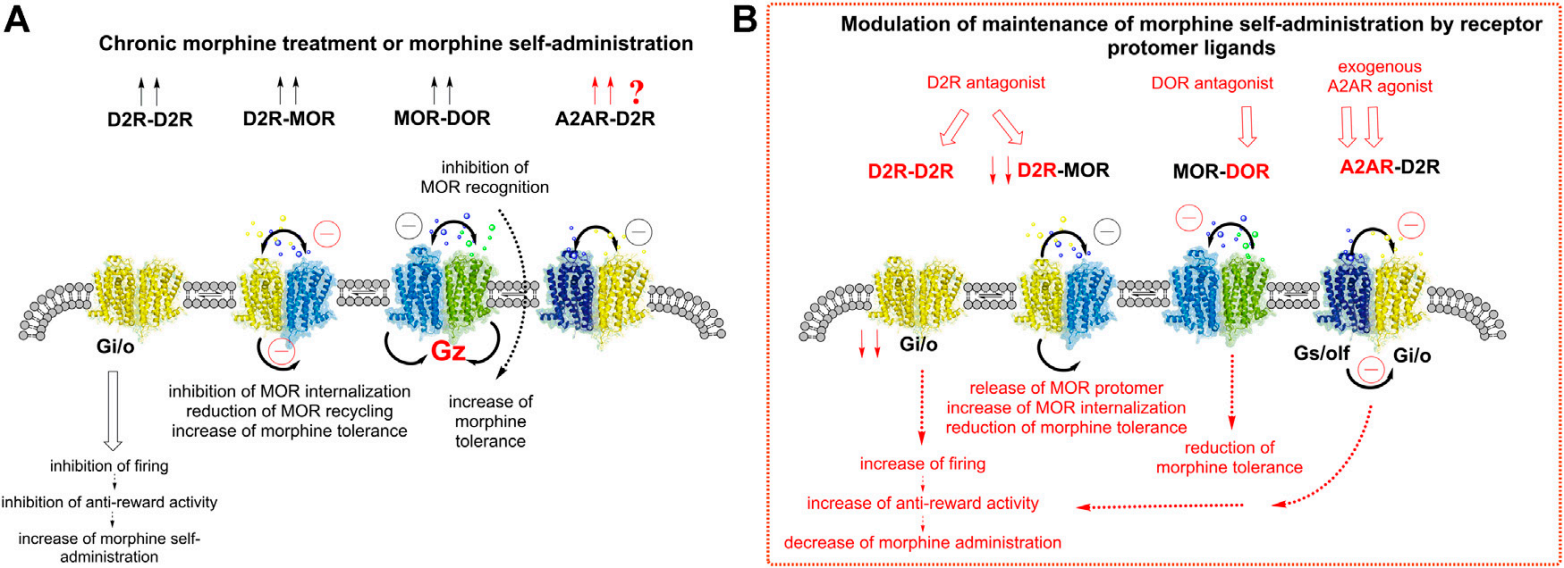
Understanding the role of the A2AR-D2R heteroreceptor complexes in modulating the changes in the activity of the ventral striatal-pallidal GABA antireward neurons induced by chronic morphine treatment or morphine self-administration. **(A)** Morphine (chronic) is shown to increase the density of D2R-D2R homomers (2 arrows pointing upwards). This causes enhanced inhibition of firing in the antireward neurons leading to a reduction of antireward activity. An increase of morphine self-administration is found. The D2R-MOR complex also becomes increased in density after morphine (chronic) mainly due to inhibition MOR internalization. This event will result in a reduction of MOR cycling, which impairs its signaling, and a reduction of recognition also develops due to antagonistic allosteric receptor-receptor interactions. As a result, an increase in morphine tolerance takes place since higher doses of morphine are needed to induce reward due to the malfunction of the MOR signaling. It is also proposed that an increased density of A2AR-D2R complexes develops upon exposure to chronic morphine as seen from the two red arrows indicated. As a result, the D2R function becomes reduced through an allosteric brake on D2R signaling. The MOR-DOR complex is well known to be increased in density upon chronic morphine treatment or morphine self-administration, shown by 2 arrows. Both receptor protomers remain functional by coupling to Gz protein. However, the morphine activation of the DOR protomer is known to produce a brake on MOR recognition via an allosteric receptor-receptor interaction. Therefore, an increase in morphine tolerance takes place. **(B)** Modulation of morphine effects by receptor protomer ligands. The D2R antagonist is known to block the inhibitory D2R homomer signaling over Gi/o and produces a marked reduction of morphine self-administration. In the case of the D2R-MOR complex, the D2R antagonist appears to disrupt the complex and set the MOR protomer free from D2R mediated allosteric inhibition. MOR internalization is therefore increased and its function restored, leading to a reduction of morphine tolerance due to enhanced MOR signaling induced by morphine. It is indicated that the A2AR agonist given *in vivo* should effectively inhibit the function of the D2R protomer of the A2AR-D2R complex, increased in density (see **A**). Thus, a reduction of the inhibitory Gi/o activity of the D2R protomer develops which results in an increase in the activity of the anti-reward GABA neurons. A reduction of morphine self-administration should develop. The DOR antagonist can target the DOR protomer and remove its allosteric inhibition of the MOR signaling, which reduces morphine tolerance.

In view of the existence of many D2R-MOR heteroreceptor complexes in the ventral striatal-pallidal GABA antireward neurons and their receptor-receptor interactions, it is suggested that they can participate in the modulation of maintenance of morphine self-administration by their receptor protomer activation or inhibition. These two receptors are both coupled to Gi/o proteins and under such conditions the two receptor protomers often interact through antagonistic receptor-receptor interactions (Harfstrand and Fuxe, 1987). In fact, blockade of D2Rs diminishes morphine tolerance in the spinal cord of the mouse (Dai et al., 2016). This implies that in the D2R-MOR complex of these neurons, D2R activation can reduce the MOR internalization and signaling of this complex to avoid excessive inhibition of the anti-reward neurons. In cellular models there also exists evidence that the D2R in this complex can reduce the speed of internalization of MOR by a MOR agonist (Vasudevan et al., 2019). Thus, it may be that the MOR protomer may stay longer in the plasma membrane but with reduced coupling to Gi/o, leading to reduced MOR signaling (Figure 2).

It is also likely that MOR-DOR heteromers exist in the GABA antireward neurons since several techniques have demonstrated high densities of these receptor heteromers inter alia in the nucleus accumbens and dorsal striatum, especially after chronic morphine treatment, which increases their formation (Gupta et al., 2010; Erbs et al., 2016) (Figure 2). Bidirectional antagonistic allosteric receptor-receptor interactions between the MOR and DOR protomers have also been demonstrated using an agonist or antagonist for one of the protomers (Gomes et al., 2000; Gomes et al., 2004; Gomes et al., 2011). It was also postulated that the MOR-DOR heteromer no longer signals via the Gi/o proteins since pertussis toxin did not prevent its signaling. It was therefore postulated that this receptor heteromer signals by recruiting the Gz subunit (George et al., 2000). This subunit can be recruited inter alia to the MOR-DOR complex via e.g., morphine treatment, is not down-regulated by chronic treatment with morphine (Kabli et al., 2014) and can be involved in producing morphine use disorder. Furthermore, a MOR-DOR-biased agonist would reduce tolerance as well as physical dependence (Gomes et al., 2013; Portoghese et al., 2017) (Figure 2B).

Naloxone is a competitive MOR antagonist (Nakamura et al., 2020) and known to block MORs leading to reduction of opioid reward and MOR-induced psychological dependence (Nakamura et al., 2020; Reeves et al., 2020). Thus, as indicated in Figure 2, the mechanism in this process can involve the blockade of the MOR protomers in the D2R-MOR and MOR-DOR heterocomplexes located in a postjunctional position in the ventral striato-pallidal GABA anti-reward neurons as well as of MOR monomers/homomers (not shown). As a result of the naloxone-induced blockade of MOR induced inhibition of the GABA antireward neurons, the activity of these GABA antireward neurons is increased which reduces the reward impact of activity in the accumbal GABA reward neurons (Everitt et al., 2008; Koob and Le Moal, 2008).

In addition, it should also be considered that MOR exist also in the type 2 vesicular glutamate transporter positive glutamate neurons modulating opioid reward at the glutamate synapses (Reeves et al., 2020). It therefore seems possible that the cortico-accumbal glutamate neurons that drive the GABA anti-reward neurons possess MOR in their synapses. Thus, MOR may inhibit the GABA anti-reward neurons in two ways, one through inhibition of glutamate release onto these neurons and another through postjunctional inhibition of activity in the GABA anti-reward neurons. Naloxone should therefore be highly efficient in increasing activity in these GABA antireward neurons, leading to a reduction of the impact of the accumbal GABA reward neurons in the emotional circuits (Everitt et al., 2008; Koob and Le Moal, 2008). An advantage of the D2R-MOR heteroreceptor complex operating via Gi/o coupling (Figure 2) is that in this case the naloxone induced blockade of the MOR also removes the antagonistic reciprocal allosteric receptor-receptor interactions in this heterocomplex. Therefore, the inhibitory signaling of the D2R can even become increased helping to maintain a certain degree of increase in the GABA antireward neurons. It is not clear to which degree the DOR protomer operating via Gi/o can take over inhibitory function of the MOR protomer upon treatment with naloxone (Stockton and Devi, 2012; Derouiche and Massotte, 2019).

Furthermore, we should consider the role of large numbers of A2AR-D2R heteroreceptor complexes in the ventral striatal-pallidal GABA antireward neurons. It is suggested that a major mechanism involved could be the A2AR agonist induced activation of the A2AR protomer in the A2AR-D2R complex, leading to enhanced inhibition of the D2R recognition and signaling. Co-injection of an A2AR agonist CGS21680 and morphine for 11 days significantly reduced morphine self-administration. However, if instead the adenosine receptor antagonist DMPX was given prior to morphine infusions, a significant increase in morphine self-administration was observed (Sahraei et al., 1999). Since neither an adenosine A1 receptor (A1R) agonist nor an A1R antagonist altered the morphine self-administration (Sahraei et al., 1999), the conclusion was that the A2AR antagonist properties of DMPX led to the expression and/or development of morphine reinforcement. As a result of the antagonistic allosteric receptor-receptor interaction in the A2AR-D2R heteroreceptor complexes, the activation of the A2A protomer results in a brake of the D2R protomer. Therefore, a reduction of the inhibitory action by the D2R on the firing of the ventral striatal-pallidal GABA antireward neurons pathway can no longer be in operation and antireward activity is increased and morphine self-administration is reduced (Figure 2).

Based on the above, it seems possible that morphine self-administration is associated with an increase in the density of D2R-MOR, MOR-DOR heteroreceptor complexes and D2R homoreceptor complexes in the ventral striatal-pallidal GABA antireward neurons (Gomes et al., 2004; Gupta et al., 2010; Dai et al., 2016; Derouiche and Massotte, 2019) (Figure 2A). We now propose that this also holds true for A2A-D2R heteroreceptor complexes in view of the previous evidence obtained in a cocaine self-administration rat model (Pintsuk et al., 2016; Borroto-Escuela et al., 2017b). Thus, it was found that cocaine self-administration produced increases in the A2A-D2R heteroreceptor complexes in the nucleus accumbens with an enhancement of their antagonistic allosteric receptor-receptor interaction. Therefore, a higher contribution of the A2AR-D2R heteroreceptor complexes results in an increase in the activity in the GABA antireward neurons. This proposed mechanism can explain the inhibitory effects of A2AR agonists on morphine self-administration (Sahraei et al., 1999) and may contribute to reducing morphine use disorder.

Based on the reorganization of the homo- and heteroreceptor complexes described above, we should consider the altered modulation of the maintenance of morphine self-administration induced by the receptor protomer ligands of these complexes (Figure 2B). The D2R antagonist, targeting the D2R homomer, will produce an activation of the anti-reward neurons by removal of the inhibitory action of the D2R on the firing of the GABA anti-reward neurons. On the other hand, D2R antagonists will disrupt the D2R-MOR complex and set the MOR protomer free from the D2R protomer. MOR internalization is therefore increased and its function restored, leading to a reduction of morphine tolerance due to enhanced MOR signaling induced by morphine. It is also indicated that an A2AR agonist given *in vivo* should effectively inhibit the function of the D2R protomer of the A2AR-D2R complex, which will be expected to increase in density (see Figure 2A). Thus, a reduction of the inhibitory Gi/o activity of the D2R protomer develops, which results in an increase in the activity of the anti-reward GABA neurons. A reduction of morphine self-administration should develop. As to the DOR antagonist, it will enhance MOR signaling by removal of the DOR protomer induced inhibitory allosteric modulation of the MOR protomer function. In this way it would reduce morphine tolerance by increasing MOR recognition and signalling (Gomes et al., 2000; Borroto-Escuela et al., 2013c; Gomes et al., 2013; Portoghese et al., 2017; Derouiche and Massotte, 2019).

## On the Role of the Dynamic Balance of MOR and D2R Heteroreceptor Complexes for Morphine Use Disorder Development

The mechanism for the development of hypersensitive MOR in morphine withdrawal in the ventral striatal-pallidal GABA neurons is unknown. It may, however, be speculated that a compensatory increase in the formation of MOR homomers takes place in morphine withdrawal. As a result, the density of D2R-MOR complexes becomes reduced due to increased formation of hypersensitive MOR homoreceptor complexes. As a result, the density of the D2R-MOR complexes will be reduced since hypersensitive MOR may develop an increased affinity for each other and formation of MOR homoreceptor complexes becomes favored. More D2Rs will therefore be available to bind to A2ARs. This leads to increased formation of A2AR-D2R complexes with enhanced antagonistic A2AR-D2R interactions, which was in fact indicated from the pharmacological analysis. Thus, such a change in the balance between the D2R-MOR, MOR-MOR and A2AR-D2R in morphine withdrawal can also contribute to the development of morphine use disorder. The D2R being under increased allosteric A2AR inhibition can no longer by itself effectively inhibit the activity of the ventral striatal-pallidal GABA antireward neurons.

The major way to reduce anti-reward activity in these GABA neurons in morphine withdrawal is through agonist activation of the hypersensitive MOR involving MOR homoreceptor complexes with morphine or other opioids or enkephalins. This MOR activation leads to inhibition of the antireward neurons.

As discussed above, after chronic morphine treatment there is a marked increase in MOR-DOR complexes in the limbic brain circuits including also the ventral striatal-pallidal GABA antireward neurons (Gupta et al., 2010; Kabli et al., 2014). Instead, in morphine withdrawal, hypersensitive MORs develop which may enhance their affinity for each other and cause an increased formation of MOR-MOR homoreceptor complexes.

This hypothesis on the role of the dynamic balance of A2AR-D2R, D2R-MOR, MOR-MOR and MOR-DOR complexes in the GABA antireward neurons for morphine use disorder development opens up a new approach for understanding and treatment of this brain disease. Like in cocaine use disorder (Borroto-Escuela et al., 2018a), it becomes important to reduce the postulated increased formation of the A2A-D2R heteroreceptor complexes also in morphine use disorder in view of the A2AR brake on D2R signaling causing antireward and aversion. A2AR antagonists can be of help for treatment of morphine use disorder, but like in cocaine use disorder treatment, the design of interface interfering peptides and/or hetero-bivalent compounds with D2R agonist and A2AR antagonist pharmacophors may be necessary to develop (Borroto-Escuela et al., 2018a). By setting the D2R free from the A2AR brake, morphine activation of MOR is no longer the only way to bring down activity in the GABA antireward pathway and morphine use disorder should go away or be reduced.

In the scenario where one didn’t develop morphine use disorder yet but still under a morphine chronic treatment, the goal of pharmacotherapy is instead to reduce its use. As in the case of cocaine use, A2AR agonist treatment can be of help (Listos et al., 2008; Borroto-Escuela et al., 2018a). By reducing D2R signaling in the A2AR-D2R complex in the GABA antireward neurons through the antagonistic allosteric A2AR-D2R interaction the inhibition of the GABA antireward neurons will be reduced. The morphine induced activation of the MOR in these neurons will therefore not be as effective in reducing the firing of the GABA antireward pathway. The rewarding effects of morphine will therefore become weakened.

## On the Role of Enkephalins in the Ventral Striatal-Pallidal GABA Antireward Neurons in Modulating the Balance of Their MOR and D2R Heteroreceptor Complexes

The MOR and DOR exist in the GABA antireward neurons of the nucleus accumbens and can be activated by enkephalins released from these neurons (Mansour et al., 1995; Mongi-Bragato et al., 2018) involving their soma-dendrites and local collaterals followed by short distance volume transmission (Fuxe et al., 2005). The work of Mongi-Bragato and colleagues (Mongi-Bragato et al., 2016) has clearly demonstrated that the enkephalins play a major role in producing cocaine sensitization at the behavioral and molecular level. They also concluded that the enkephalins can be key players in mediating psychostimulant addiction like cocaine addiction by contributing to the changes in neuronal plasticity with increases in AMPA receptors, phosphorylation of tyrosine receptor kinase B and CREB causing the addiction development (Mongi-Bragato et al., 2018). These findings are of high interest and knockout of the proenkephalin gene abolished the development of cocaine sensitization as did treatment with the opioid receptor antagonist naloxone (Mongi-Bragato et al., 2016).

Based on the current hypothesis on the role of the balance of the A2AR-D2R, MOR-DOR, MOR-D2R, and MOR-MOR complexes in the GABA antireward neurons for the development of morphine use disorder the work by Mongi-Bragato and colleagues (Mongi-Bragato et al., 2016; Mongi-Bragato et al., 2018) implies that this balance is of relevance also for cocaine use disorder. The postulated increase, in the brake of the inhibitory D2R protomer signaling in the A2AR-D2R-Sigma1R complex in the enkephalin positive GABA anti-reward neurons in cocaine use (Borroto-Escuela et al., 2018a), may to begin with be compensated for by extracellular enkephalin release, activating the MOR-DOR complex. Increased met-enkephalin levels have been observed in nucleus accumbens upon cocaine treatment. Thus, via its Gi/o and/or Gαz coupling the MOR-DOR signaling may increase the inhibition of the activity of the GABA anti-reward neurons. However, cocaine use disorder may develop when the MOR-DOR and MOR-MOR complexes are no longer able to cause sufficient inhibition of the GABA anti-reward neurons due to an irreversible and permanent brake on the D2R signaling in the A2AR-D2R-Sigma1R complex and/or insufficient release of enkephalins from these neurons. Thus, the balance of the D2R-MOR, MOR-DOR and A2AR-D2R heteroreceptor complexes as well as of MOR-MOR homoreceptor complexes in the enkephalin positive GABA antireward neurons appears to be critical for understanding both morphine and cocaine use disorders.

## Conclusion

Establishing the role of the balance of D2R-MOR, MOR-DOR and A2AR-D2R heteroreceptor complexes, including their corresponding homoreceptor complexes, in the GABA antireward neurons appears to be of high relevance for understanding the molecular basis of morphine and cocaine use disorder. Therefore, we should consider these receptor protomers as new targets for novel treatments of these brain diseases. Patients suffering from morphine dependence can become more dependent on morphine actions at MOR protomers in MOR-DOR and MOR-MOR complexes in the antireward GABA neurons in view of increased expression of A2AR-D2R complexes antagonizing inhibitory D2R signaling. As a consequence, morphine may produce inhibition of these neurons mainly through the activation of the Gi/o and Galphaz mediated inhibitory signaling of MOR in a receptor complex with DOR. The integration of signaling in D2R-MOR complex remains to be determined but is proposed to be reduced in density in morphine withdrawal due to a postulated increase in hypersensitive MOR-MOR homoreceptor complexes. Overall, the activation of the A2AR-D2R complex in the ventral striatal-pallidal GABA antireward neurons by favoring antireward and aversion may reduce morphine induced reward produced via activation of the MOR homo- and heteroreceptor complexes, inhibiting activity in these antireward neurons.
